# Molecular screening and docking analysis of LMTK3and AKT1 combined inhibitors

**DOI:** 10.6026/97320630014499

**Published:** 2018-12-09

**Authors:** Loubna Allam, Ghrifi Fatima, Lakhlili Wiame, El Amri Hamid, Ibrahim Azeddine

**Affiliations:** 1 Biotechnology Laboratory (Medbiotech), BioInova Research center, Rabat Medical and Pharmacy School, MedBiotech Center,Mohammed V University in Rabat, Rabat, 10000, Morroco; 2 Genetics Laboratory of Royal Gendarmery, Rabat, Morocco. Loubna Allam

**Keywords:** AKT1, QSAR, LMTK3, Virtual screening, ATP-inhibitors

## Abstract

The abnormal activation of AKT/mTOR signaling pathway and overexpression of LMTK3, are the main factors involved in the generation of drug resistance. Therefore, the use of computer-aided drug design in the inhibitors discovery offers an advantage to provide new candidates for the treatment of this resistance. We realised the virtual screening and molecular docking of AKT1 and LMTK3 proteins by the Dockblaster server. In addition, with abundance of candidates under development for AKT1 kinase, we have also conducted a Quantitative Structure-Activity Relationship (QSAR) study based on these compounds, in order to design more active compounds and predict their activity for development of a new inhibitor of AKT1. QSAR tests were performed for AKT1 using the Partial Least Squares method with a correlation coefficient of R2=0.8062 and a cross-validation of q2=0.6995. This test has selected five compounds as competitive inhibitors-AKT1-ATP with a better biological activities. In parallel the molecular screening has selected five other compounds as competitive ATP-inhibitors of LMTK3. One of them is a common inhibitor with AKT1, and it is marketed as a moderate to severe pain therapy. The ADME predictions confirmed the inhibitors pharmacological activity of these compounds for potential consideration as drug candidates.

## Background

Breast cancer is a disease characterized by the accumulation of several molecular alterations causing tumors with specific phenotypes 1. The abnormal activation of AKT/mTOR signaling pathway and the overexpression of LMTK3, are the main factors involved in the induction of resistance 2,3. These factors constitute therefore a very interesting field of research. This is why LMTK3 and AKT have been considered among the promising therapeutic targets in the treatment of different cancers 2-4. LMTK3 is incriminated in induction of resistance to hormonal treatment by two mechanisms. Firstly, it regulates of ESR1 transcriptional activation by inhibiting PKC, which leads to decrease of phosphorylated AKT (Ser473), responsible for FOXO3 stabilization which allows the increase of ERa transcriptional activity 2. Secondly, it allows the ERa protein direct phosphorylation, protect it from protease degradation mediated by ubiquitin (Ub) 2. The aberrant expression of LMTK3 and AKT has been observed in several types of human's cancer 2-6. However, recent studies have demonstrated that inhibition of LMTK3 2, and pathway (AKT and/or mTOR) deletion, restore sensitivity to anti-estrogen treatment in breast cancer cells 4. These proteins can therefore, play a critical role in the cancer therapy development 5,6, AKT1 and LMTK3 have characteristics necessary for design of effective and reliable small drug molecule. The reason we opted in this study to predict molecule able to provoke simultaneous inhibition of these both proteins. This inhibition will be a crucial step in the combined treatment of diseases characterized by abnormal signaling of AKT1 and LMTK3. In the present study, we realised the virtual screening and the molecular docking study of the AKT1 and LMTK3 proteins by the DockBlaster server to highlight new molecules on the basis of their ability to interact with the kinase. We developed a Quantitative Structure Activity Relationship Study (QSAR) to provide structural information in order to design and propose more active compounds and develop new AKT1 inhibitors.

## Methodology

### Softwares:

All softwares were open acces, except the academic version of MOE (2008.10) obtained from Chemical Computing Group (CCP) (Monreal, QC, Canada).

### Dataset and QSAR study:

A set of 341 molecules with inhibitory activity for AKT1 was selected from the CanSAR database 7 (https://cansar.icr.ac.uk/) based on their molecular weight and IC50. This was converted to pIC50 for the QSAR analysis. The set of these molecules was randomly distributed to a training set of 240 compounds (70% of the data) and a test set of 101 compounds (30% of the data). The training set has been subjected to the Partial Least Square (PLS) 8. In absence of an experimentally validated inhibitor with a biological activity of LMTK3 protein in the different databases, the construction of QSAR model based on the biological activity of these inibitors cannot take place.

### Data preparation and descriptor selection:

More than 184 bidimentionnels descriptors (2D) in Molecular Operating Environment (MOE) were calculated 8. The calculated descriptors were screened for their invariant nature by the statistical application QuaSAR-Contingency module of MOE. 47 molecular descriptors were selected by intercorrelation between all of them, than were used in the QSAR model construction.

### Generation of QSAR model:

We used the Partial Least Square (PLS) as a statistical analysis method to establish a linear correlation between the subset of descriptors and bioactivities to derive predictive models. These models were established on a training set (240 molecules), and tested by test set (101 molecules). The module QuaSAR-Model in MOE was used to construct the QSAR PLS model 8.

### Model Validation:

The QSAR model obtained has been validated in two steps. The first step through internal validation by allowing the calculation of the cross correlation coefficient (q2), using the LOO (cross validation Leave-One-Out). The second step through external validation used to evaluate the prediction set activities and calculating the numerical model parameters.

### Virtual screening:

The LMTK3 and AKT1 proteins 3D structures in active form 'in coformation' obtained respectively, one by homology modeling approach (reported in our previous study) 9, and the other extracted from PDB database(ID:3OCB). The ATP binding cavity of both 3D structures was used as targets for the virtual screening. After Virtual screening by Dock Blaster serv(http://blaster.docking.org), the ligands with the best energy score were selected. The visualization of docking results by DOCK 3.6 software 10 was analyzed using PyMol software. The developed QSAR model of AKT1 protein was used to calculate the predicted activity of 100 selected compounds of AKT1 extracted.

### ADME prediction and toxicity:

In order to verify the lead candidates, pharmacokinetic properties selected, ADME predictions were realized by SwissADME server (http://www.swissadme.ch). Six important properties of oral bioavailability are calculated. Each property has an optimal values range and it is fraction Csp3 > 0.25 for saturation. The TPSA must be between 20 and 130 Å 11. For solubility, the logS (calculated with the ESOL model 12) must not exceed 6. The lipophily, XLOGP3 13 must be between -0.7 and +6.0, and for flexibility, the molecule must not have more than 9 rotative bonds.

## Results

### QSAR Analysis:

**Figure 1:**
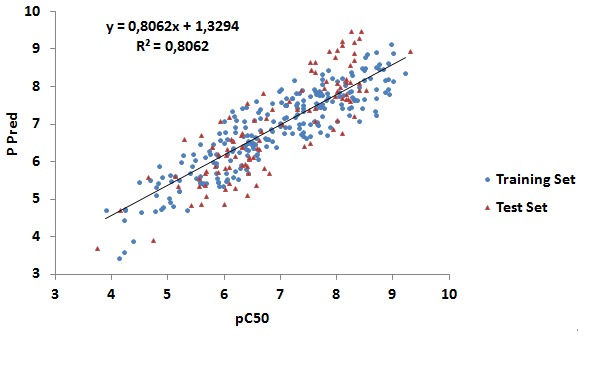
Relationship between observed and predicted data from QSAR model. Notes: the compounds of the model QSAR are represented by dots, those of training set are in blue and those of test set are in red. Abbreviations: Pred, Predict; QSAR, quantitative structure-activity relationship.

The QSAR model of AKT1 was constructed on the 47 molecular descriptors basis. After the QSAR model regression analysis of 240molecules, a QSAR model with a correlation coefficient (R2) of 0.8062 and a RMSE of 0.532 was obtainedas shown in Figure 1. Parameters R2, q2 are used to select the best QSAR model, which has been validated and evaluated by a Leave-One-Out (LOO) cross validation method. The predictive performances of this model were represented by cross-validated RMSE with 0.66699, and cross-validated R2 with 0.69958.The QSAR model of AKT1 protein validated was used to filter a set of 100 chemical structures, extracted from the DockBlaster server. We selected 74 compounds with activity predictive value greater than 6 14.

### Virtual screening:

**Figure 2:**
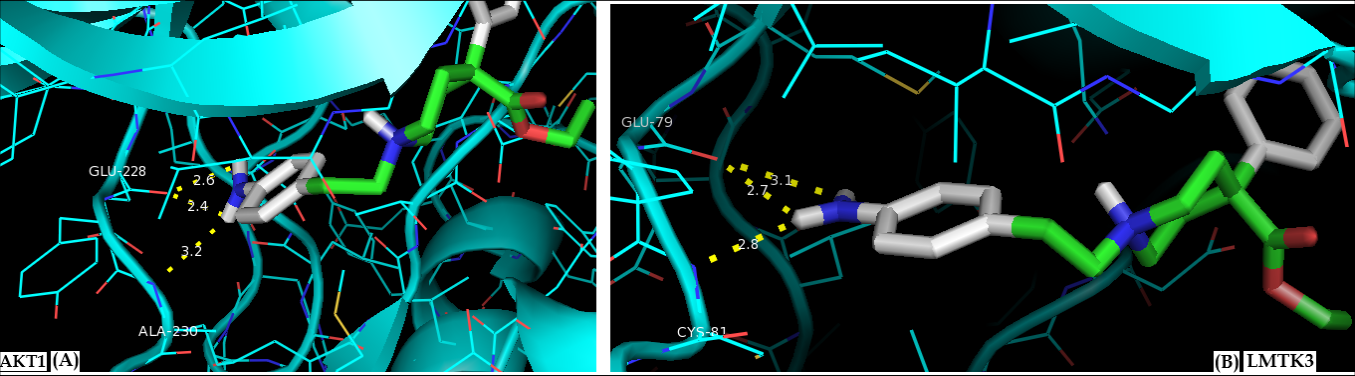
Visualization of different interactions between the compound which inhibits both AKT1 and LMTK3 through hydrogen bonds and its localization in the active site of both proteins. The identical compound (numbered n°33 for AKT1 and n�21 for LMK3) interacts with both proteins through three hydrogen bonds of their hinge region of the catalytic site. (A) AKT1 ; (B) LMTK3. Notes: Red areas mean oxygen atom, blue areas mean nitrogen atom, and green areas mean other. The dotted yellow line represents the bonds. The numbers represent the size of the hydrogen bonds established between the ligand and the receptor.

After molecular docking, the best results are those that have low energy hydrogen bonds between the ligand and the active site of each target (LMTK3-in or AKT1-in), as shown in Table 1. The five best compounds obtained for each protein have respectively an affinity between -8.6 and -7.8 kcal/mol for LMTK3 and between - 8.3 and -7.2 (pC50> 6) for AKT1. We obtained a common compound that binds at a time on the LMTK3 active site and the AKT1 active site. It realizes respectively, three interactions with the hinge region of the catalytic site of AKT1 (Compound n°33) as illustrated in Figure 2 (A), and three hydrogen bonds with the hinge region of the catalytic site of LMTK3 (Compound n°21) in Figure 2 (B).

### ADME analysis:

The ADME property of all lead candidates selected for LMTK3 and SwissADME server analyzed AKT1. The values obtained of TPSA were comprised between 20 and 130Å, the logS (calculated with the ESOL model did not exceed the value 6). While XLOGP3 values were all less than 6.0. For flexibility, all the molecules had less than 9 rotatable bonds. Most of the molecules had a Csp3 greater than 0.25, with a negative result for the compounds n°25, 41 and 55 selected for AKT1, due to their simple architecture. This architecture gives an advantage to scientists to explore the more diverse chemical space depending on the shape of the active site. The bioavailability of all investigated molecules for LMTK3 and AKT1 is fully included in the valid area.

## Discussion

In this study we were able to establish a good correlation between the biological activity and the properties related to the structure in the proposed QSAR model, which leaves thought that this model provides good predictive ability and offers a useful alternative for predicting the biological activity of untested molecules. This study is therefore considered to be one of the first QSAR studies as than of Ajmani et al. 15. These studies make it possible to estimate the biological activity of molecules that can be potent inhibitors of AKT1. Ajmani et al. 15 used a set of AKT1 inhibitors available in various literature reports. Their developed QSAR model has a predicted correlation coefficient (R2 pred) of 0.689a nd a "Leave-One-Out" correlation value (q2) of 0.628. For this purpose and to better understand the AKT1 inhibition structural requirements, we have in the present study, to increase the structurally varied inhibitors number, collected from the canSAR database. The latter provides detailed scientific information on cancer and gathers billions of experimental measurements, thus giving the best quantitative model with a "Leave-One-Out" correlation value (q2) of 0.5316, and R2 pred of 0.8062. After virtual screening and interaction mode analysis of selected compounds with both proteins. Five compounds were interesting for each target. A compound in common has been selected. It binds simultaneously at site of LMTK3 and at that of AKT1, respectively, with an affinity of -7.9 and -8.3kcal/mol.

Visualization of these interactions showed that there are, three hydrogen bonds with AKT1 side chains in Glu228 and Ala230, and three bonds with LMTK3 side chains in Gly79 and Cys81, which are considered key residues of their hinges. Indeed, the other compounds also interact with the hinge of their protein. The affinities are comprised between -7.9 and -7.1 kcal/mol for LMTK3, and between -8.1 and -7.5kcal/mol for AKT1.

The pharmacokinetic analysis of these main candidates showed that These compounds are soluble or medium-soluble in water, with high gastrointestinal absorption, they may also be capable to cross the blood-brain barrier 16. All of these molecules are flexible with less than 9 rotary links. In the together; predictions of ADME confirmed the pharmacokinetic properties of the five lead candidates selected for AKT1 and those selected for LMTK3.

In their study, Anbarasu and Jayanthi 17 were the first to suggest new inhibitors of inactive form of LMTK3 (out conformation). These inhibitors were not fixed on ATP site of our model developed in the previous study 9 (data not shown). This can be explained by the difference in conformation of two models developed, and consequently, the location difference of ATP binding site in both studies. These same researchers completed their studies on LMTK3-out 18. They were able to identify derivatives of curucumin as potential inhibitors of LMTK3-out.

For this purpose, we judged useful to develop other specific and selective inhibitors of the ATP binding site of active form of LMTK3 to complement those developed by Anabarasu et al. of inactive form of LMTK3. Our study will also support further studies such as the Piedrafita et al. project, which aims to discover potent and selective inhibitors of LMTK3 that will be used to pharmacologically validate LMTK3 as a new target for Breast Cancer treatments.

Thus, we hypothesized that double inhibition of LMTK3 and AKT1 proteins is a combination therapy with antitumor activity. Therefore, these are new therapeutic targets that can stimulate the development of new cancer treatment strategies. Our next study will be the search for the efficiency of the association of these small molecules, which inhibit LMTK3 and AKT1 in cancer by in vitro and in vivo tests. If successful, this study will have a significant translational impact, potentially adding new kinase inhibitors to the set of cancer drugs.

## Conclusion

The methods used in this study will allow the design new inhibitors. They will be more selective with better structural features and a potent and specific anti-cancer activity enhanced for AKT1 and LMTK3. The compounds selected by molecular screening for LMTK3 and AKT1 may provide a starting point for new ATP-competitive inhibitory classes of these two proteins. Encouraging results have been obtained with the identification of a drug candidate as a combined inhibitor of LMTK3 and AKT1. This molecule has an analgesic effect. It has been tested and validated in vivo and marketed as a therapeutic molecule to treat moderate to severe pain. That's why, in the next study, we will test this compound directly on cancer cell lines.

## Figures and Tables

**Table 1:** Docked interaction analysis of different ligands screened with the two proteins (LMTK3-in and AKT1-in).

Ligands number	Receptor (Protein)	Number of H-bonds	Active site residues	Number of interacting bonds	Bonds length in Å
					
25ZINC48769846	AKT1	7	Ala230 (Hinge)	2	2.80 - 2.85
			THR291	1	2.93
			Asp292	4	2.21 -2.76 - 2.92 - 2.93
70ZINC83364573	AKT1	8	Ala230 (Hinge)	1	2.53
			TYR229 (Hinge)	1	3.07
			GLY230 (Hinge)	1	3.29
			ASN231	1	3.41
			Asp292	4	2.58 - 3.06 - 3.64 - 3.77
41ZINC83373478	AKT1	9	Glu228 (Hinge)	1	2.42
			Ala230 (Hinge)	1	2.4
			LYS158	1	2.44
			Asp292	6	2.23-2.26-3.29-3.41-3.46-3.87
55ZINC83373686	AKT1	7	Glu228 (Hinge)	3	2.32-3.31-3.75
			Ala230 (Hinge)	1	2.99
			THR291	1	3.26
			Asp292	2	2.39-2.5
33 Drug has analgesic effect	AKT1	3	GLU228 (hinge)	2	2.4-2.6
			ALA230 (hinge	1	3.2
21 Drug has analgesic effect	LMTK3	3	GLU79 (hinge)	2	2.7-3.1
			CYS81 (hinge)	1	2.8
18Zinc3830224	LMTK3	4	CYS81 (hinge)	2	3.0-3.0
			ASP152	1	2.7
			LYS32	1	3.4
98 Zinc3831014	LMTK3	2	CYS81 (hinge)	2	2.9-3.2
71Zinc8551669	LMTK3	4	CYS81 (hinge)	2	2.3-2.8
			ASN139	1	2.8
			ASP152	1	3
15 Zinc30612467	LMTK3	4	GLU79 (hinge)	1	3
			CYS81 (hinge)	1	2.9
			ARG138	1	2.1
			ASN139	1	3.5

## Abbreviations

AKT1: AKT serine/threonine kinase 1
LMTK3: Lemur Tyrosine Kinase 3
3D: Three-Dimensional
QSAR: Quantitative Structure Activity Relationship
2D: Two-Dimensional
PLS: Partial Least Square
PDB: Protein Data Base
TPSA: Topological Surface Area
Pred: Predict
